# *Paracoccidioides lutzii* Plp43 Is an Active Glucanase with Partial Antigenic Identity with *P. brasiliensis* gp43

**DOI:** 10.1371/journal.pntd.0003111

**Published:** 2014-08-28

**Authors:** Natanael P. Leitão, Milene C. Vallejo, Palloma M. Conceição, Zoilo P. Camargo, Rosane Hahn, Rosana Puccia

**Affiliations:** 1 Departamento de Microbiologia, Imunologia e Parasitologia, Escola Paulista de Medicina-Universidade Federal de São Paulo (EPM-UNIFESP), São Paulo, Brazil; 2 Núcleo de Doenças Infecciosas e Tropicais de Mato Grosso, Universidade Federal de Mato Grosso, Mato Grosso, Brazil; University of California San Diego School of Medicine, United States of America

## Abstract

**Background:**

*Paracoccidioides brasiliensis* and *P. lutzii* cause paracoccidioidomycosis (PCM). *P. brasiliensis* main diagnostic antigen is glycoprotein gp43, and its peptide sequence is 81% identical with a *P. lutzii* ortholog here called Plp43. *P. lutzii* (“Pb01-like”) apparently predominates in Midwestern/Northern Brazil, where high percentages of false-negative reactions using *P. brasiliensis* antigens have recently been reported. The aim of this work was to produce recombinant Plp43 to study its antigenic identity with gp43.

**Methodology:**

We expressed rPlp43 as a secreted major component in *Pichia pastoris* and studied its reactivity in immunoblot with PCM patients' sera from Southwestern and Midwestern Brazil.

**Principal Findings:**

We showed that rPlp43 is not glycosylated and bears glucanase activity. The protein did not react with anti-gp43 monoclonal antibodies in immunoblot, suggesting absence of the corresponding gp43 epitopes. Nevertheless, common epitope(s) might exist, considering that gp43-positive PCM sera recognized rPlp43 in immunoblot, while gp43-negative sera (33 out of 51) from patients resident in Midwestern Brazil were also rPlp43-negative. Two genotyped *P. lutzii* were from patients with gp43-negative sera, suggesting that non-reactive sera are from patients infected with this species.

**Conclusion:**

Our data suggest that gp43 and Plp43 bear one or only a few common epitopes and that gp43 cannot be used in diagnosis of PCM patients infected with *P. lutzii* probably because Plp43 is poorly expressed during infection.

## Author Summary

*Paracoccidioides lutzii* is currently recognized as a distinct species from *P. brasiliensis* that until then was solely responsible for paracoccidioidomycosis (PCM). Serological diagnostic of this primary systemic mycosis is quite sensitive due to the expression of *P. brasiliensis* antigen gp43, which has specific peptide epitopes recognized by patients' sera. Unexpected PCM false-negative reactions have recently been reported for patients from Midwestern/Northern Brazil, where *P. lutzii* (“Pb01-like”) apparently predominates. In our work, 33 PCM false-negative sera from Midwestern Brazil, including two cases of *P. lutzii* genotyped isolates, did not recognize recombinant Plp43 (gp43 ortholog from Pb01) or gp43 (native purified or recombinant), suggesting Plp43 is possibly underexpressed in vivo. Gp43-positive sera reacted with rPl43 showing that common epitopes do exist, although anti-gp43 MAbs did not recognize the orthologous *P. lutzii* protein. The main finding of our work is that gp43 cannot be used in diagnosis of PCM patients infected with *P. lutzii*.

## Introduction

Until recently, all fungal isolates causing paracoccidioidomycosis (PCM) were considered to be from a single species named *Paracoccidioides brasiliensis*. Multilocus studies including 65 clinical and environmental isolates from Latin American countries showed that *P. brasiliensis* is a complex of at least three phylogenetic species: S1 (species 1), the most numerous; PS3 (phylogenetic species 3), composed of clonal samples from Colombia; and PS2 that is phylogenetically cryptic and groups six isolates bearing the most polymorphic genes [1]. Later studies including samples from Midwestern and Northern Brazil showed that 17 isolates formed a more distant clade from S1/PS3/PS2 [2]. They have the same genetic profile as Pb01, which has been highly used as model in the *Paracoccidioides* literature [3]. Independent and concatenated genealogies derived from the concordance method of phylogenetic species recognition supported the “Pb01-like” clade as a new phylogenetic species [2], recently recognized a distinct species, named *P. lutzii*, based not only on phylogeny, but also on comparative genomics [4], recombination analysis, and morphological features [5].

*P. brasiliensis* and *P. lutzii* are temperature-dependent dimorphic fungi and PCM is a primary granulomatous systemic mycosis, whose infection occurs by inhalation of fungal conidia produced by environmental mycelia [5]. Infectious particles transform into the pathogenic yeast form in the pulmonary alveolar epithelium, where infection starts, but the yeasts can then spread to other body sites. Active pulmonary or lymphatic PCM forms occur in up to 2% of infected individuals, who can reach 10 million throughout endemic areas of Latin America [6]. Differences in clinical manifestations related to the *Paracoccidioides* species are suggested, but that kind of association needs further investigation [5].

Mycological diagnosis based on the finding of fungal species identified in clinical specimens is still gold standard for mycoses. In PCM caused by *P. brasiliensis*, serological diagnosis and follow up are also useful and accurate tools used by clinicians [7]. This is due to the expression of fungal glycoprotein gp43, which is a major antigen bearing species-specific peptide epitopes [8]. The Pb*GP43* gene is composed of two exons encoding 416 amino acids that include a 35-residue signal peptide [9]. The translated protein is structurally related to beta-1,3-glucanases, however the expressed protein in culture medium is inactive [9]. Processed extracellular gp43 contains a single *N*-glycosylation site that is occupied by a high-mannose chain bearing 13 to 14 non-phosphorylated mannose residues and one terminal β-D-galactofuranose [10]. This sugar chain can be removed enzymatically with endoglycosydase H (Endo H), generating a faster migrating band in SDS-PAGE gels [11]. Besides other types of interaction with the immune system [12] the molecule also contains a well-studied T-cell epitope (P10) that elicits protective cellular immunity in experimentally infected animals and can be used in immunotherapy in association with standard chemotherapy [13]–[15]. Gp43 is secreted as clusters through defined regions of the cell wall [16]. Surface gp43 is partially responsible for adherence of yeast cells to components of the extracellular matrix [17], like laminin and fibronectin, in adhesion assays with Vero cells [18].

Gp43 has been tested in numerous serological assays [7], [8], [12] and spontaneous anti-gp43 idiotype antibodies that could be useful in PCM diagnosis were also found in PCM patients [19]. The antigen is highly specific for PCM caused by *P. brasiliensis* when the immunoassays preserve its natural conformation or eliminate sugar epitopes [8], [20]–[22]. That is true for both native and recombinant gp43 produced in *Pichia pastoris* [21], which can be purified from culture supernatants using affinity columns coupled with anti-gp43 monoclonal antibody [23].

The most used test for PCM diagnosis is double immunodiffusion (I.D.) because of its simplicity, high sensitivity, and specificity [7]. Whole *P. brasiliensis* preparations, generally from yeast-form culture supernatants, are commonly used as antigen. In these preparations, gp43 is the antigenic component responsible for PCM specificity and positivity above 80% [24], [25]. False-negative reactions have been attributed to intense pulmonary infection, immune depression, and to low affinity antibodies recognizing carbohydrate epitopes [24], [26]. The Pb*GP43* gene is highly polymorphic at exon 2 and the most informative in multilocus studies [1], [2], [27]. As a consequence, gp43 has isoforms varying in isoelectric point (pIs) according with the genotype group [27], [ reviewed in 28]. Experimental evidence with recombinant gp43 shows, however, that most PCM sera can recognize different *P. brasiliensis* isoforms [21]. That is probably due to one common epitope that is specifically recognized by MAb17c [29].

*P. lutzii* (“Pb01-like”) apparently predominates in Midwestern Brazil [5], where high percentages of false-negative I.D. reactions using *P. brasiliensis* antigens have recently been reported [30]. That suggests that gp43 is not recognized by sera from patients living in those areas. Since gp43 peptide sequence is only 81% identical with the closest *P. lutzii* ortholog, here called Plp43 (PAAG_05770 at http://www.broadinstitute.org/annotation/genome/paracoccidioides_brasiliensis/MultiHome.html), the aim of this work was to produce recombinant Plp43 and to study its immunological identity with gp43. We followed this strategy because we could not detect Plp43 in Pb01 extracts using anti-gp43 antibodies. Therefore, we could not purify or characterize native Plp43. Our preliminary results thus suggested that either Plp43 was expressed in Pb01 at undetectable amounts in our culture conditions or it showed substantial antigenic differences to prevent recognition by anti-gp43 monoclonal and polyclonal antibodies. By working with recombinant Plp43 produced in *Pichia pastoris* we observed that the molecule is not glycosylated and that it preserves glucanase activity. We tested the recombinant protein with a panel of PCM sera from proved cases, besides anti-gp43 MAbs. Our data suggest that gp43 and Plp43 bear one or only a few common epitopes and that gp43 cannot be used in diagnosis of PCM patients infected with *P. lutzii* probably because Plp43 is poorly expressed during infection.

## Materials and Methods

### Sera

Anti-gp43 monoclonal antibodies have previously been obtained in our laboratory [11]. PCM sera were obtained from patients showing varied clinical forms and that were either untreated or under treatment. Sera from Brazilian Midwest (51 samples) were from Dr. Rosane Hahn's collection (UFMT, Cuiabá, Brazil). The corresponding patients were diagnosed with PCM using mycological methods of visualizing the fungus in clinical samples. Sera from Brazilian Southeast (50 samples) were from Dr. Zoilo Pires de Camargo's collection (UNIFESP, São Paulo, Brazil); positivity in I.D. serological tests denoted PCM. This study was approved by the Research Ethics Committee with protocol numbers 0366/07; 1796/10 by Universidade Federal de São Paulo and CAAE: 17177613.6.0000.5541 by the Federal University of Mato Grosso (UFMT). All patient samples were anonymized.

### Synthesis of Plp43 cDNA and Expression in Bacteria and *P. pastoris*

**Figure 1:**
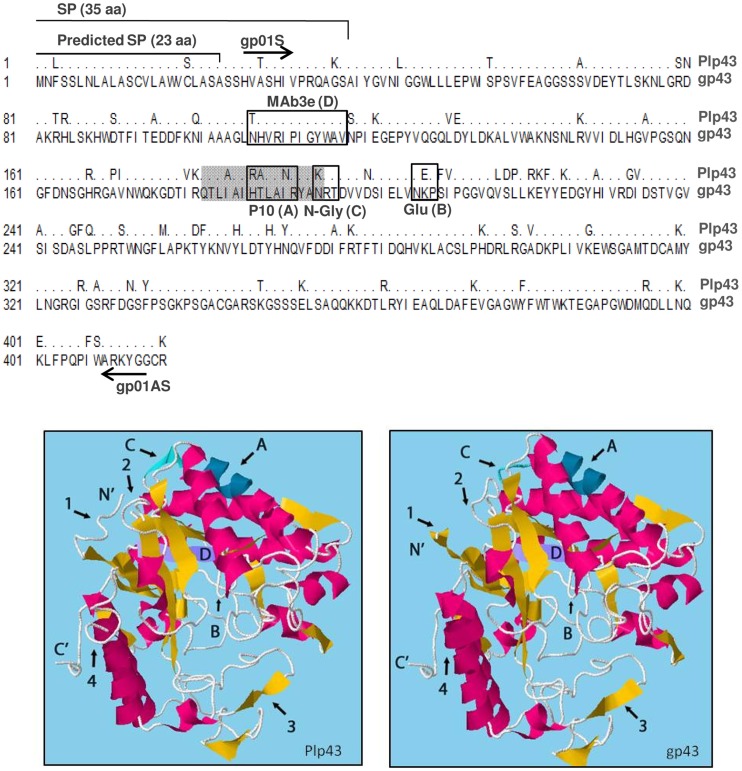
Amino acid sequence alignment and molecular models show differences between Plp43 (from Pb01) and gp43 (from Pb18). Differences in amino acid residues can be seen. Known sequence features are indicated: SP (signal peptide), MAb3e epitope (D/purple), T-cell P10 epitope (grey shade with boxed antigenic core; A/navy), N-glycosylation site (N-gly, C/green), and NEP glucanase active site (Glu, B). The sequences corresponding to primers gp01S and gp01AS used to amplify Pl*P43* ORF are shown. In the molecular models below, arrows (1–4) point to remarkable differences between Plp43 and gp43; caps letters indicate the motifs above specified. Yellow, beta-sheets; magenta, alpha-helix.

A fragment corresponding to Plp43 cDNA (1,167 kb) devoid of 84 bp from *N*-terminal was reverse transcribed from DNA-free total RNA purified from *P. lutzii* Pb01. Total RNA extraction was performed in the presence of TRIzol reagent (Invitrogen), according with the manufacturer's recommendations and our previous report [21], from yeast cells cultivated at 36°C, with shaking, in mYPD (0.5% yeast extract, 0.5% peptone-casein, and 1.5% glucose, pH 6.5). Pl*P43* cDNA was obtained by reverse transcription-PCR (RT-PCR) using the ThermoScript RT-PCR system (Gibco). Specific antisense primer gp01AS (5′- GGTACCTCAATCCACCATACTTCCTA-3′), that has a 5′ *Kpn*I cleavage site (Fig. 1), was used to obtain the first strand. The second strand of the open reading frame was elongated under standard PCR conditions with the sense primer gp01S (5′-GTCGACAGGCAAAGTCAGCAATA-3′), containing a *Sal*I site, which anneals immediately downstream a predicted signal peptide.

The PCR fragment was purified after electrophoresis from agarose gel using the QIAEXII kit (Qiagen), and then cloned into pGEM-T Easy vector (Promega) for maintenance. The cloned fragment was sequenced to confirm ORF identity and subcloned into the *Sal*I/*Kpn*I sites of the bacterial expression vector pHIS1 [31]. The recombinant protein was expressed in *Escherichia coli* BL21 pLysS genotipe F^−^ cytoplasm with a His_6_ tag to facilitate purification in a nickel column.

To obtain recombinant Plp43 in a eukaryotic system, the insert was excised from pHIS1 and subcloned into pPIC9 (Invitrogen) *Eco*R1/*Not*1 sites for expression in *Pichia pastoris*. *P. pastoris* was also transformed with pPIC9 alone (empty vector, EV) to be used as negative control. To verify *P. pastoris* heterologous protein production, random individual colonies were grown for two days, with shaking at 30°C, in BMGY (buffered minimal glycerol complex medium), composed of 1% yeast extract, 2% peptone, 0.1 M potassium phosphate, pH 6.0, 1.34% YNB (Difco), 1% ammonium sulfate, and 1% glycerol. Cell pellets were suspended in one-half the initial volume of BMMY (BMGY with 1% methanol instead of glycerol) and incubated at 30°C under agitation for 3 days. Inducing methanol (1%) was added daily and expression of rPlp43 was verified in stained SDS-PAGE profiles of culture supernatants from *P. pastoris* clones. *P. pastoris* EV was used as negative control. The best-producing clone was selected for large-scale production of rPlp43.

### Immunodiffusion (I.D.)

I.D. tests [32] were carried out only with PCM patients' sera from Brazilian Midwest using purified gp43 (200 ng) as antigen. The assays were performed as described in our previous report [21], and the results from overnight reactions were recorded after staining precipitation bands in dried agarose with Coomasie brilliant blue (CBB).

### SDS-PAGE and Western Blotting

Protein profile analysis was obtained by SDS-PAGE and polyacrylamide gel staining with CBB [33]. For Western blotting reactions, the samples were transferred to nitrocellulose membranes [34], which were then incubated with patients' sera (1∶1,000) or anti-gp43 mouse monoclonal antibodies (MAb) (10 µg/ml). Immunocomplexes were evidenced with goat anti-human and anti-mouse, IgG-peroxidase conjugated (Sigma) and developed by chemiluminescence with luminol (ECL reagent, Pierce).

### Enzymatic Deglycosylation

Enzymatic deglycosylation of glycoproteins was carried out with Endo H (endo-β-*N*-acetylglucosaminidase, Sigma), which removes high mannose *N*-linked chains, according with manufactures' recommendations. Approximately 1 µg of protein was incubated with reaction buffer (250 mM NaH_2_PO_4_, pH 5.5) and denaturing solution (2% SDS and 1 M β-mercaptoethanol) at 100°C for 5 min. After cooling, 2 U of enzyme were added for overnight incubation at 37°C. The reaction products were analyzed in stained SDS-PAGE.

### Genotyping *P. lutzii* Isolates

We had two paired samples of patients' gp43-negative sera and the corresponding fungal isolate samples. In order to genotype the *Paracoccidioides* isolates to ascertain they corresponded to *P. lutzii* species as hypothesized, fungal DNA was extracted [35] and used in PCR reactions with primers that anneal to a specific region of the *HSP70* gene only present in *P. lutzii* (HSPMM1, 5′-AACCAACCCCCTCTGTCTTG-3′, and HSPMM2, 5′-TACCCTGTTCGTTGGCAATG-3′), as described [3]. DNA from *P. lutzii* Pb01 was used as positive control and from *P. brasiliensis* Pb18 as negative control.

### Exoglucanase Activity

Enzymatic exoglucanase activity was performed according with Conchie et al. [36]. Methanol-induced *P. pastoris* culture supernatants were dialyzed against 50 mM acetate buffer, pH 5.5, and incubated with PNPG substrate (p-nitrophenyl-β-glucoside) (Sigma) at 37°C, for 4 h. The reactions were interrupted by addition of 0.37 M Na_2_CO_3_ and color was recorded at *A*_420_. Culture supernatants from *S. cerevisiae* S288, *P. pastoris* 339 wild type and *P. pastoris* EV growing under agitation at 30°C for 24 h were used as positive controls. BSA and native gp43 [9] were used as negative controls.

### Modeling

Tertiary protein structure model was created using Raptor X server (http://raptorx.uchicago.edu) [37], a protein threading program that predicts tertiary polypeptide structure based on alignment sequence and function scores, that estimate the fitness of a sequence-structure alignment. Protein sequences corresponding to Pb18 gp43 and Pb01 Plp43 without signal peptide (35 aminoacids) were analyzed and main epitopes or relevant proteins regions were highlighted.

### Ethics Statement

This study was approved by the Research Ethics Committee with protocol numbers 0366/07; 1796/10 by Universidade Federal de São Paulo and CAAE: 17177613.6.0000.5541 by the Federal University of Mato Grosso (UFMT). All patient samples were anonymized.

## Results

### Plp43 Sequence Features and Heterologous Expression

According to the *Paracoccidioides* genome data at the Broad Institute site (http://www.broadinstitute.org/annotation/genome/paracoccidioides_brasiliensis/MultiHome.html), the closest gp43 homologous sequence from Pb01 (PAAG_05770) bears 416 amino acids translated from a 1,251-long ORF. The DNA sequence has a predicted intron of 76 residues, while in *P. brasiliensis* the gp43 intron is 78-bp long [9]. The Pb01 peptide sequence is 81% identical to Pb18 gp43. In the alignment seen in Figure 1, note that the signal peptide is conserved and predicted to be 23-residue long. Nevertheless, *N*-terminal sequencing of purified gp43 suggested that it expands 35 residues [9]. In that case, the secreted proteins have a predicted molecular mass of about 42 kDa; however the Pb01 sequence has a pI value of 9.4, while in *P. brasiliensis* the calculated gp43 pI values vary between 6.8 and 8.5 [28], showing important amino acid shifts. In the molecular model, the Plp43 *N*-terminus is linear, in contrast to the gp43 beta sheet found in the same region. Number 4 denotes a similar change, but closer to the *C*-terminus. In 2 and 3 there is a spin in the secondary structure.

Figure 1 also shows that the *N*-glycosylation site is absent from Pb01 Plp43, while the glucanase NEP site is preserved, thus suggesting that the protein is not *N*-glycosylated and possibly retains full enzymatic activity, as opposed to gp43 [9]. Therefore, we called the gp43 ortholog in *P. lutzii* Plp43. Also note in the molecular model that the *N*-glycosylation region is linear in gp43, but forms a beta-sheet in Plp43. The P10 epitope shows enough alterations in its core to theoretically impair Plp43 recognition by T-cells [13]. On the other hand, the gp43 MAb3e epitope is mostly conserved.

**Figure 2:**
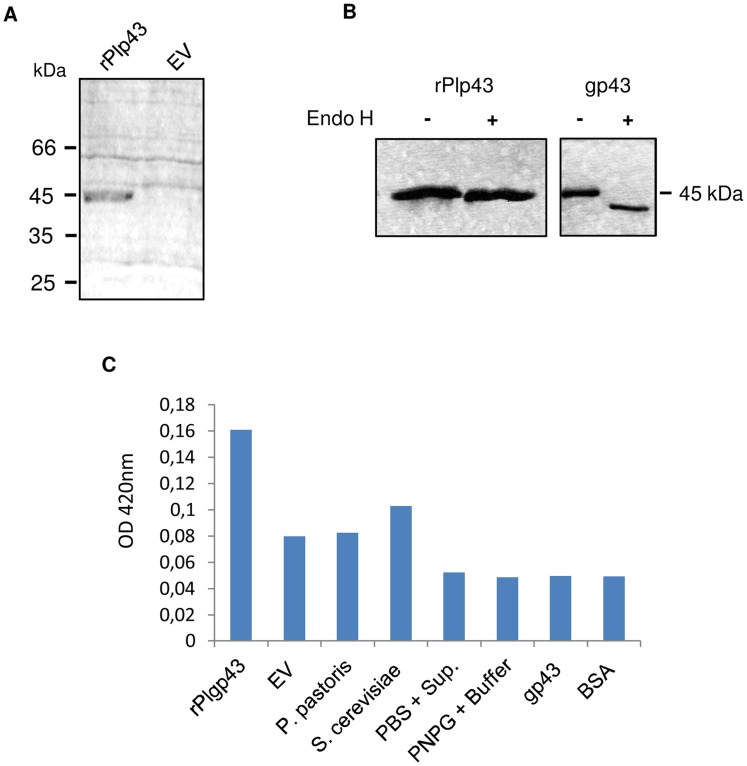
Recombinant rPlp43 is not glycosylated and seems to be enzymatically active. (**A**) Heterologous expression of rPlp43 in *P. pastoris* culture supernatants from methanol-induced recombinant yeasts containing the Pl*P43* insert or not (EV, empty vector). (**B**) SDS-PAGE profile of rPlp43 and control gp43 before (−) and after (+) treatment with Endo H. (**C**) Glucanase activity against PNPG in culture supernatants from *P. pastoris* expressing rPlp43 when compared with that, at equivalent total protein amount, of yeasts containing empty vector (EV), wild type *P. pastoris* and *S. cerevisiae*. Purified gp43 was used as protein negative control at equivalent protein amount to rPlp43, as estimated in SDS-PAGE gels. BSA was also included as negative control at higher amounts. PBS+rPlp43 (without substrate) and PNPG+enzyme buffer (without substrate) were used as negative controls. Migration of standard molecular masses is indicated.

Previous unpublished results in our lab suggested that Plp43 was either unexpressed in Pb01 within our in vitro conditions or undetectable with anti-gp43 polyclonal antibodies. Therefore, due to the consequent difficulties to work with native Plp43, we expressed the corresponding Pl*P43* gene in prokaryotic and eukaryotic systems (Figure 2) in order to study the protein antigenic and enzymatic features.

For Plp43 heterologous expression, we used total RNA isolated from *P. lutzii* Pb01 in RT-PCR reactions with specific primers that allowed elongation of processed Plp43 lacking the first 25 *N*-terminal residues and two *C*-terminal residues (Figure 1). The corresponding PCR fragment was cloned into pGEM-T, sequenced, and subcloned into pHIS1 [31] and pPIC9 (Invitrogen) vectors for expression in bacteria and in *P. pastoris*, respectively. Expression of Plp43 in *E. coli* Bl21 pLysS resulted in intracellular expression of an approximately 45-kD insoluble protein (data not shown). The recombinant molecule was enriched in a Ni-Sepharose 6 fast flow column under urea-denaturing conditions, used in preliminary immunological assays and also to obtain anti-Plp43 polyclonal rabbit serum.

Plp43 expression in *P. pastoris* induced for three days in methanol resulted in a major secreted component (rPlp43) in culture supernatants migrating at 45 kDa (Figure 2A). Due to cloning procedures detailed in Materials and Methods, rPlp43 has three amino acid residues translated from pHIS1 nucleotides remaining at the 5′ end.

As previously mentioned, translated Plp43 does not have any predicted *N*-glycosylation site in the processed molecule, since the NRT motif in gp43 is altered to KRT (Figure 1). We confirmed that Plp43 is not glycosylated by treating rPlp43 with Endo H: note in Figure 2B that both treated and untreated rPlp43 migrated equally in SDS-PAGE, while control native gp43 (or *P. pastoris* recombinant gp43r3, not shown), migrated faster due to the cleavage of a high-mannose chain [11], [21]. Migration of rPlp43 and gp43 was similar probably because rPlp43 has extra amino acid residues resulting from cloning procedures.

We also tested if Plp43 is an active glucanase by assaying rPlp43-containing *P. pastoris* culture supernatants for enzymatic activity. Figure 2C highly suggests that rPlp43 is indeed an active exo-glucanase, considering that an equivalent protein amount of culture supernatant from methanol-induced *P. pastoris* harboring an empty vector was 50% less active against PNPG.

### Reactivity of Recombinant rPlp43 with Anti-MAbs

**Figure 3:**
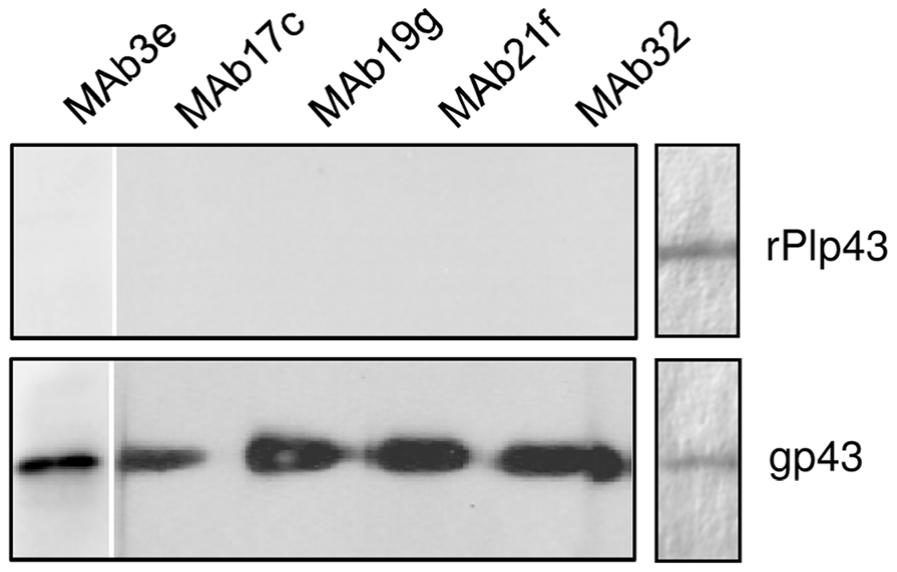
Immunoblot reactivity of anti-gp43 monoclonal antibodies with gp43 and rPlp43. Reactivity of rPlp43 was tested with five different anti-gp43 MAbs (indicated) in immunoblot, using gp43 as positive control. On the right, CBB-stained SDS-PAGE gel showing the amount of antigen used in the assay. The results were the same for HisPlp43 expressed as inclusion bodies in bacteria.

We tested both HisPlp43 (not shown) and rPlp43 for reactivity with anti-gp43 MAbs in immunoblotting (Figure 3) and did not observe any reactivity with MAb3e, Mab17c, Mab19g, MAb21f or MAb32 (11, 34). These results suggested that the epitopes recognized by these MAbs are not present in Plp43. MAb17c is used in affinity columns for gp43 purification [29]; however, due to the above results it could not be used to purify rPlp43. Therefore, we used *P. pastoris* culture supernatants containing rPlp43 (and not the purified protein) in the following experiments. We chose to work further only with rPlp43 because it is expressed in a soluble form in culture supernatants, therefore matching the soluble controls of native gp43 or *P. pastoris* gp43r3.

### Reactivity of rPlp43 with PCM Patients' Sera

We tested the rPlp43 reactivity in immunoblot with PCM patients' sera from Midwestern (M) and Southern (S) Brazil (Table S1). Sera from Southwestern Brazil (50 total) were I.D.-positive (1∶2 to 1∶32 or higher) against total Pb339 antigen (Table S2) containing high amounts of gp43 as major component [20], [38]. The assays were performed for either PCM diagnosis or treatment follow up at Dr. Camargo's laboratory. Sera from Midwestern Brazil (51 total) were from patients confirmed for PCM with direct microscopy, however they were I.D.-negative at Dr. Hahn's laboratory. These I.D. assays were carried out with similar Pb339 total antigen preparations mentioned above [20]. We then re-evaluated these sera using I.D. with known amounts of purified gp43, which allows for better sensitivity than culture supernatant preparations [21]. A total of 16 out of 51 were then I.D.-positive (pure to 1∶32) against purified gp43 (). The 33 sera from Midwestern PCM patients that were I.D.-negative (51M to 79M) became the focus of the present work, because they apparently did not recognize gp43.

**Figure 4:**
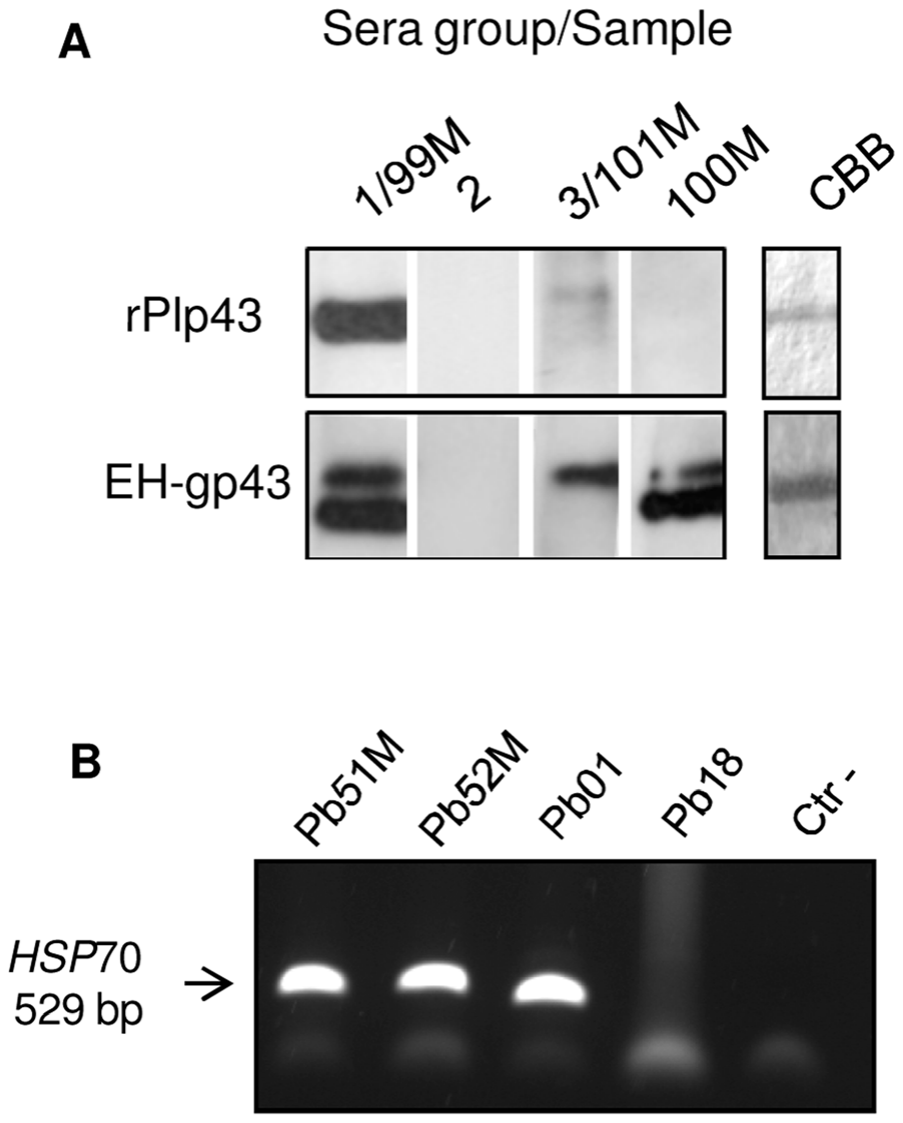
(A) Representative immunoblot reactions of PCM patients' sera (1∶1,000) with rPlp43 and Endo H-deglycosylated (EH-) gp43. The overall results are shown in Table 1 for sera groups 1, 2, 3, and individual sera 99M, 100M, and101M. On the right, CBB-stained SDS-PAGE gel showing the amount of antigen used in the reactions. Results with Endo H-deglycosylated gp43r3 were similar to (EH-)gp43 and are not shown. (**B**) **PCR amplification of** *HSP70* **using primers for the** *P. lutzii* **gene as shown in an agarose gel.** Partial *HSP70* PCR (529 bp) amplification shows that clinical isolates Pb51M and Pb52M are *P. lutzii*. DNA extracted from Pb01 (*P. lutzii*) and Pb18 (*P. brasiliensis*) were used as species control. Crt-, in the absence of DNA.

In order to prove this hypothesis, we tested all sera listed on Table S1 using chemiluminescence immunoblot, at a single dilution of 1∶1,000, in order to achieve the highest possible sensitivity. As antigen, we used rPlp43 (in *P. pastoris* culture supernatant); we added controls of purified native gp43 and recombinant gp43r3 from *P. pastoris* [21] treated or not with Endo H. Upon treatment with Endo H, a large percentage of gp43 is deglycosylated, but generally not 100% [11]. Therefore, we were able to visualize reactivity with both forms, since deglycosylated gp43 migrates faster at about 38 kDa. However, previously to using Endo H-treated antigen, all sera were tested with both gp43 and gp43r3. Figure 4A shows the types of data we obtained and Table 1 summarizes the results. Sera from group 1 and serum 99M showed strong bands with both glycosylated and deglycosylated gp43 and gp43r3, as expected from their positive I.D. reactions. They also reacted strongly with rPlp43. Serum 100M had similar profile, except for the lack of reactivity with rPlp43, suggesting that the gp43 epitopes recognized by this serum are absent in Plp43. Sera from group 2, all of them I.D.-negative from Midwestern Brazil, did not react with any tested antigen. Four sera (group 3 and 101M) were only reactive with glycosylated gp43 and gp43r3, suggesting cross-reactivity with carbohydrate epitopes, which are not specific for PCM and are also absent in Plp43. Therefore, all ID-positive sera reacted with deglycosylated gp43, which proved the specificity of the reaction (serum 99M was an exception), and also with rPlp43 (with exception of 95M and 99M). That suggested that one or more gp43 peptide epitopes are present in Plp43. On the other hand, ID-negative sera did not recognize gp43 or rPlp43 peptide epitopes in a sensitive immunoblot reaction. Serum 99M was an exception probably because I.D. was not sensitive enough to detect low amounts of antibody. Considering that the so far scare literature data point to high concentration of *P. lutzii* in Midwestern Brazil [5], our results suggest that the I.D.-negative patients were infected with *P. lutzii*. We believe that this is the case based on two Midwestern gp43-negative serum samples (51M and 52M) for which we had the corresponding fungal isolates collected from the respective patients. The *Paracoccidioides* cultures isolated from these patients (Pb51M and Pb52M) were genotyped as *P. lutzii* using PCR (Figure 4B) of a *HSP70* fragment [3], thus supporting our working hypothesis. In addition, Plp43 might be poorly expressed during infection with *P. lutzii*, considering that none of the I.D.-negative sera reacted with Plp43.

**Table 1:** Overall results of the I.D. and immunoblot reactivity of recombinant *P. lutzii* (rPlp43), native or recombinant gp43 (gp43, gp43r3) and their Endo H-deglycosylated forms (EH-gp43, EH-gp43r3) with PCM patients' sera from Brazilian Southeastern (S) and Midwestern (M) regions (Table S1).

Group* or serum	ID#	rPlp43	Gp43 or gp43r3	EH-gp43 or EH-gp43r3
**1**	+	+	+	+
**2**	−	−	−	−
**3**	−	−	+	−
**99M**	−	+	+	+
**100M**	+	−	+	+
**101M**	+	−	+	−

*Sera groups: 1, 1S–50S and 82M–95M; 2, 51M–81M; 3, 96M–98M.

# ID titers with purified gp43 or Pb339 culture supernantant are specified in Table S2.

## Discussion

We presently expressed Plp43, which is the closest *P. lutzii* ortholog of *P. brasiliensis* main diagnostic antigen gp43 [8]. We showed evidence that gp43 cannot be used in serology of PCM caused by *P. lutzii* because it is not recognized by sera of patients likely to be infected with this species. The same sera did not react with recombinant Plp43 either, suggesting it is under expressed during infection. Although Plp43 peptide sequence is 81% identical with gp43, it has key differences resulting from polymorphism in characteristic gp43 motifs. The NRT *N*-glycosylated site is mutated to KRT in translated Plp43 and we here experimentally confirmed that the recombinant protein is not *N*-glycosylated when expressed in yeast. We also observed that the NEP glucanase site is preserved in Plp43 and our results suggest that it is an active glucanase, as opposed to gp43 [9]. In addition, we tested the rPlp43 ability to bind to fibronectin in affinity-ligand assays and at least one gp43 motif responsible for fibronectin binding is apparently preserved in rPlp43 (data not shown). Glycoprotein gp43 is mostly responsible for *P. brasiliensis* binding to Vero cells and both laminin and fibronectin are involved in this process [18]. Four gp43 peptides were able to inhibit binding at different percentages, but the highest inhibition (57%) was achieved with NLGRDAKRHL. When comparing gp43 and Plp43 sequences, only peptides that inhibited binding at percentages lower than 30% were conserved, specifically, SAQQKKDTLRYI (91,7% identical in Plp43) and ITEDDFKNIA (80% identical in Plp43).

The calculated pI for mature Plp43 is 9.5, while in gp43 isoforms the pIs vary between 6.37 and 8.35 [27]. Orthologous mature glucanase sequences from *H. capsulatum* (HCAG_04584) and *B. dermatitidis* (BDFG_01423) have molecular masses around 43 kDa and are 72% identical to Plp43. Their pIs are also basic, about 8.5–8.7. On the other hand, the corresponding orthologs from *Aspergillus niger* (XP_001398868.1) and *A. fumigatus* (Afu1g03600), *C. albicans* (CAWG_01085), *P. pastoris* (XP_002491361.1), and *Trycophyton tonsurans* (EGD99595.1), all of them with preserved NEP active sites, are acidic (pIs 4.5 to 5.0), but with similar molecular mass (44 kDa). A phylogenetic tree shows clear separation between the basic/neutral and the acidic sequence clades (data not shown).

Our main goal was to assay Plp43 reactivity with PCM patients' antibodies in comparison with gp43. This is a key type of information in PCM diagnosis of individuals infected with *P. lutzii*. Epitopes recognized by anti-gp43 MAbs 3e, 19f, 21f, 17c, and 32 [11], [29] were not detected in Plp43 when HisPlp43 and rPlp43 were tested in immunoblot. Nevertheless, at least one common epitope is likely to exist, considering that gp43-positive PCM sera also reacted with rPlp43, however less intensely. On the other hand, gp43-negative sera were also rPlp43-negative. These sera came from patients resident in Midwestern Brazil, where *P. lutzii* is apparently prevalent [5]. Indeed, two fungal samples corresponding to gp43/Plp43-negative sera were genotyped as *P. lutzii* (Figure 4B). The Brazilian Midwestern sample we presently analyzed was composed of 51 PCM sera. If our assumption is correct, about 69% (anti-gp43 negative sera), are from *P. lutzii*-infected patients, thus corroborating the phylogenetic studies showing prevalence of the species in that particular endemic area [5], [39]. It remains to be further tested if gp43-negative sera from PCM patients living in other endemic areas could also be from *P. lutzii*-infected patients, or if negative reactions are really due to intense pulmonary infection/immune depression or low affinity antibodies recognizing carbohydrate epitopes [24], [26]. It is worth noticing that reactions against gp43 carbohydrate epitopes cannot be considered specific to PCM because these epitopes are promiscuous among fungi [8].

We believe that Plp43 is expressed at low amounts by *P. lutzii* both *in vivo* and *in vitro*, considering that anti-HisPlp43 rabbit serum presently produced did not react with any Pb01 culture supernatant, intracellular or cell wall extract component in immunoblot (data not shown). Regulation of gp43 expression is still poorly understood and transcription levels vary with the isolate [40], [41]. We have studied the 5′ non-translated region of the Pb*GP43* gene and observed that it differs from that of the Pl*P43* gene, probably leading to distinct regulation and lower transcription.

In conclusion, our data suggested that although there is at least one common epitope between gp43 and Plp43, gp43 cannot be used in diagnosis of PCM patients infected with *P. lutzii* because Plp43 is apparently poorly expressed during infection.

## Supporting Information

Table S1 PCM patients' sera assayed in this work showing the original numbers and the codes presently attributed to them (S, Southern; M, Midwestern Brazil).
(DOCX)

Table S2 I.D. titers of PCM patients' sera listed in Table 1. Titers for sera 51M to 99M were presently obtained with purified gp43. Titers for sera 1S to 50S were attributed by the time of diagnosis with Pb339 culture supernatant preparations.
(DOCX)
