# Supplementary material for: Paracoccidioides lutzii Plp43 Is an Active Glucanase with Partial Antigenic Identity with P. brasiliensis gp43
Source: PLoS Negl Trop Dis. 2014 Aug 28;8(8):e3111. doi: 10.1371/journal.pntd.0003111 (PMC4148228; doi:10.1371/journal.pntd.0003111)
Supplement: Table S1 — PCM patients' sera assayed in this work showing the original numbers and the codes presently attributed to them (S, Southern; M, Midwestern Brazil). (DOCX) [file pntd.0003111.s001.docx]

**Table S1.** **PCM patients’ sera assayed in this work showing the original numbers and the codes presently attributed to them (S, Southern; M, Midwestern Brazil).**

| **code** | original number | **code** | original number | **code** | original number | **code** | original number |
| --- | --- | --- | --- | --- | --- | --- | --- |
|  |  |  |  |  |  |  |  |
| **1S** | 3 | **27S** | 1853 | **53M** | 3 | **79M** | 151 |
| **2S** | 6 | **28S** | 1875 | **54M** | 4 | **80M** | 164 |
| **3S** | 12 | **29S** | 1921 | **55M** | 10 | **81M** | 169 |
| **4S** | 479 | **30S** | 1936 | **56M** | 11 | **82M** | 82 |
| **5S** | 481 | **31S** | 1945 | **57M** | 27 | **83M** | 99 |
| **6S** | 482 | **32S** | 1974 | **58M** | 30 | **84M** | 105 |
| **7S** | 485 | **33S** | 1977 | **59M** | 63 | **85M** | 107 |
| **8S** | 487 | **34S** | 1978 | **60M** | 79 | **86M** | 113 |
| **9S** | 1209 | **35S** | 1986 | **61M** | 80 | **87M** | 152 |
| **10S** | 1265 | **36S** | 1989 | **62M** | 84 | **88M** | 157 |
| **11S** | 1328 | **37S** | 1991 | **63M** | 102 | **89M** | 158 |
| **12S** | 1364 | **38S** | 2003 | **64M** | 108 | **90M** | 17 |
| **13S** | 1365 | **39S** | 2006 | **65M** | 111 | **91M** | 23 |
| **14S** | 1404 | **40S** | 2018 | **66M** | 115 | **92M** | 32 |
| **15S** | 1413 | **41S** | 2023 | **67M** | 120 | **93M** | 38 |
| **16S** | 1467 | **42S** | 2042 | **68M** | 125 | **94M** | 49 |
| **17S** | 1491 | **43S** | 2050 | **69M** | 126 | **95M** | 74 |
| **18S** | 1522 | **44S** | 2103 | **70M** | 128 | **96M** | 91 |
| **19S** | 1538 | **45S** | 1925 | **71M** | 129 | **97M** | 98 |
| **20S** | 1560 | **46S** | 1662 | **72M** | 130 | **98M** | 170 |
| **21S** | 1676 | **47S** | 1607 | **73M** | 139 | **99M** | 122 |
| **22S** | 1586 | **48S** | 1536 | **74M** | 143 | **100M** | 137 |
| **23S** | 1603 | **49S** | 1865 | **75M** | 144 | **101M** | 154 |
| **24S** | 1655 | **50S** | 1674 | **76M** | 146 |  |  |
| **25S** | 1678 | **51M** | 175 | **77M** | 147 |  |  |
| **26S** | 1805 | **52M** | 207 | **78M** | 148 |  |  |
