# Supplementary material for: Paracoccidioides lutzii Plp43 Is an Active Glucanase with Partial Antigenic Identity with P. brasiliensis gp43
Source: PLoS Negl Trop Dis. 2014 Aug 28;8(8):e3111. doi: 10.1371/journal.pntd.0003111 (PMC4148228; doi:10.1371/journal.pntd.0003111)
Supplement: Table S2 — I.D. titers of PCM patients' sera listed in Table 1. Titers for sera 51M to 99M were presently obtained with purified gp43. Titers for sera 1S to 50S were attributed by the time of diagnosis with Pb339 culture supernatant preparations. (DOCX) [file pntd.0003111.s002.docx]

**Table S2.** ID titers of PCM patients’ sera listed in Table 1. Titers for sera 51M to 99M were presently obtained with purified gp43. Titers for sera 1S to 50S were attributed by the time of diagnosis with Pb339 culture supernatant preparations.

| **Antigen** | | |
| --- | --- | --- |
| **ID titer** | Pb339 sup. | gp43 |
| **Pure** | - | 90M, 91M, 94M, 95 M and 101M |
| **1:2** | 1S - 13S | 82M |
| **1:4** | 14S-34S | 100M |
| **1:8** | 35S – 38S | 83M-87M and 92M |
| **1:16** | 39S – 41S | - |
| **1:32 or <** | 42S – 50S | 88M, 89M, and 93M |
| **Negative** | - | 51M-81M and 96M-99M |
